# LncRNAs Landscape in the patients of primary gout by microarray analysis

**DOI:** 10.1371/journal.pone.0232918

**Published:** 2021-02-18

**Authors:** Yu-Feng Qing, Jian-Xiong Zheng, Yi-Ping Tang, Fei Dai, Zeng-Rong Dong, Quan-Bo Zhang

**Affiliations:** 1 Precision Medicine Key Laboratory of Sichuan Province & Precision Medicine Center, West China Hospital, Sichuan University, Chengdu, China; 2 Department of Rheumatology and Immunology, Affiliated Hospital of North Sichuan Medical College, Nanchong, China; 3 Department of Geriatrics, Affiliated Hospital of North Sichuan Medical College, Nanchong, China; Suez Canal University Faculty of Medicine, EGYPT

## Abstract

To determine the expression profile and clinical significance of long non-coding RNAs (lncRNAs) in peripheral blood mononuclear cells (PBMCs) of patients with primary gout and healthy control subjects. Human lncRNA microarrays were used to identify the differentially expressed lncRNAs and mRNAs in primary gout patients (n = 6) and healthy control subjects (n = 6). Bioinformatics analyses were performed to predict the roles of differently expressed lncRNAs and mRNAs. Quantitative real-time polymerase chain reaction (qRT-PCR) was performed to detect the expression levels of 8 lnRNAs in 64 primary gout patients and 32 healthy control subjects. Spearman’s correlation was used to analyze the correlation between these eight lncRNAs and the laboratory values of gout patients. A receiver operating characteristic (ROC) curve was constructed to evaluate the diagnostic value of the lncRNAs identified in gout. The microarray analysis identified 1479 differentially expressed lncRNAs (879 more highly expressed and 600 more lowly expressed), 862 differentially expressed mRNAs (390 more highly expressed and 472 more lowly expressed) in primary gout (fold change > 2, P < 0.05), respectively. The bioinformatic analysis indicated that the differentially expressed lncRNAs regulated the abnormally expressed mRNAs, which were involved in the pathogenesis of gout through several different pathways. The expression levels of TCONS_00004393 and ENST00000566457 were significantly increased in the acute gout flare group than those in the intercritical gout group or healthy subjects (P<0.01). Moreover, inflammation indicators were positive correlated with TCONS_00004393 and ENST00000566457 expression levels. The areas under the ROC curve of ENST00000566457 and NR-026756 were 0.868 and 0.948, respectively. Our results provide novel insight into the mechanisms of primary gout, and reveal that TCONS_00004393 and ENST00000566457 might be as candidate targets for the treatment of gout flare; ENST00000566457 and NR-026756 could effectively discriminate between the gout and the healthy control groups.

## Introduction

Gout, the one of most common form of autoinflammatory arthritis in human, is characterized by elevated urate and monosodium urate (MSU) crystal deposition in tissues, which leads to arthritis, occurrence of soft tissue masses (i.e., tophi), nephrolithiasis, and urate nephropathy [1]. The epidemiological evidence suggests that both the incidence and prevalence of gout are rising, and the incidence is 1.14% and 1.4% in Shandong coastal cities of Eastern China and eastern counties respectively [2]. The specific pathogenesis of gout is still unclear. Previous studies have demonstrated that an acute gout flare is triggered by the deposition of MSU crystals in the joint and MSU crystals are widely recognized as an endogenous danger signal by components of the innate immune system [3, 4]. Hyperuricemia is the biochemical basis of gout [5].

Long noncoding RNAs (lncRNAs) are typified by a length of transcription longer than 200 nucleotides that are not translated into proteins [6]. Increasing scientific interest in these factors stems from previous investigations showing that lncRNAs exert regulatory effects on gene expression levels, involving epigenetic regulation, transcriptional regulation, and post-transcriptional regulation in the form of RNA [7]. LncRNAs also play important roles in modulating innate and adaptive immune responses and immune cell development [8]. Moreover, emerging evidence suggests that lncRNA could be as diagnostic biomarkers in many diseases such as some cancer [9], osteoporosis [10], Alzheimer’s disease [11], etc. Thus, lncRNAs have attracted much attention in recent medical studies.

The diagnosis of gout depends heavily on the higher concentration of serum urate. However, the concentration of serum urate was not observed increased in some acute gout flare patients [12]. In addition, few research of lncRNA in gout is reported. Therefore, the present study was initiated to use lncRNA microarray for the characterization of genome-wide lncRNA and messenger RNA (mRNA) expression profiles of primary gout patients compared with healthy control subjects. Our goal was to elucidate the role of lncRNAs in gout, provide new insights into the pathogenesis of gout and help to identify prospective targets for gout.

## Materials and methods

### Patients and sample collection

Sixty-four consecutive male patients with primary gout were enrolled from the Department of Rheumatology and Immunology of Affiliated Hospital of North Sichuan Medical College between February 2019 and September 2020. The classification of gout fulfilled the 1977 American Rheumatism Association, now the American College of Rheumatology (ACR), preliminary criteria for the classification of the acute arthritis of primary gout, as well as the 2015 ACR/European League Against Rheumatism (EULAR) gout classification criteria [13, 14]. All gout patients had no history of cancer, hematopathy, nephropathy, infection, or other autoimmune diseases. According to G-CAN consensus statement with regards to labels and definitions of disease states of gout [15], Gout patients were divided into an acute gout flare group (32 patients) and intercritical gout group (32 patients) based on whether patients are presenting onset of symptoms or not. The gout patients were not receiving any systemic anti-inflammatory treatment or drugs to control the production and elimination of serum urate before blood samples were obtained. Healthy controls (32 age-matched men) with no hyperuricemia, metabolic syndrome, or other chronic diseases were recruited from the Physical Examination Center of Affiliated Hospital of Sichuan North Medical University for participation in our comparative study.

Clinical laboratory evaluations of serum urate (sUA) level, blood glucose (GLU) level, and inflammation and lipid metabolism indicators were performed. The inflammation indicators estimated included erythrocyte sedimentation rate (ESR), high-sensitivity C-reactive protein (CRP), white blood cell counts (WBC), neutrophil granulocyte counts (GR), lymphocyte counts (LY), monocyte counts (Mo), and the lipid metabolism indicators include plasma total cholesterol (TC), triglycerides (TG), high-density lipoprotein cholesterol (HDL), low density lipoprotein cholesterol (LDL), very low-density lipoprotein (VLDL), apolipoprotein A1(apoA1) and apolipoprotein B100 (apoB100). All of the measurements were carried out by the Clinical Laboratory Department of the Affiliated Hospital of North Sichuan Medical College. The demographic and clinical features of all the subjects are summarized in Table 1. Peripheral blood anticoagulated with ethylene diamine tetraacetic (EDTA) was obtained from all subjects. Human peripheral blood mononuclear cells (PBMCs) were isolated with Ficoll-Hypaque gradients. Written informed consent was obtained from all of the enrolled participants. This study was approved by the Ethics Committee of the Affiliated Hospital, North Sichuan Medical College and conducted in accordance with the ethical guidelines of the 1975 Declaration of Helsinki.

**Table 1:** Clinical and laboratory data of subjects studied.

	Gout group (n = 64)	AG group (n = 32)	IG group (n = 32)	HC group (n = 32)
Age(years)	40.52±11.19	41.56±11.88	39.47±10.54	45.25±14.17
Gender F/M	0/64	0/32	0/32	0/32
Disease duration, median (range) (months)	42 (12–81)	42(1.5–75)	42(12–102)	-
BMI (kg/m^2^) ($\bar{x}$±SD)	25.20±3.94^a^	26.18±2.82^a^	25.89±3.03^a^	22.17±2.27
sUA (μmol/L) ($\bar{x}$±SD)	455.23±143.21^a^	466.99±131.19^a^	443.46±155.5^a^	366.11±53.42
GLU (mmol/L) ($\bar{x}$±SD)	5.56±0.96^a^	5.30±0.76	5.76±1.07^a^	4.91±0.44
WBC (×10^9^/L) ($\bar{x}$±SD)	7.72±2.82^a^	8.60±3.17^a^^b^	6.85±2.14	5.7±1.29
GR (×10^9^/L) ($\bar{x}$±SD)	4.88±2.37^a^	5.72±2.71^a^^b^	4.04±1.6	3.4±0.92
LY (×10^9^/L) ($\bar{x}$±SD)	2.13±0.78^a^	2.10±0.77	2.16±0.79^a^	1.78±0.6
Mo (×10^9^/L) ($\bar{x}$±SD)	0.49±0.21^a^	0.55±0.23^a^^b^	0.43±0.17^a^	0.33±0.09
ESR (mm/h) ($\bar{x}$±SD)	15.70±18.55	23.69±22.78^a^	7.72±6.98	-
CRP (mg/L) ($\bar{x}$±SD)	10.53±19.40	19.21±25.63^a^	2.75±2.57	-
TG (mmol/L) ($\bar{x}$±SD)	2.49±1.88^a^	1.97±1.26	2.49±2.02^a^	1.27±0.35
TC (mmol/L) ($\bar{x}$±SD)	4.66±0.82	4.34±0.73^a^	4.94±0.82^a^	4.5±0.64
HDL (mmol/L) ($\bar{x}$±SD)	1.18±0.21	1.18±0.23	1.17±0.2	1.17±0.22
LDLC (mmol/L) ($\bar{x}$±SD)	2.64±0.55	2.28±0.64^a^^b^	2.74±0.62	2.62±0.56
VLDL (mmol/L) ($\bar{x}$±SD)	0.88±0.35^a^	0.85±0.53	0.93±0.42^a^	0.68±0.19
apoA1 (mmol/L) ($\bar{x}$±SD)	1.10±0.19^a^	1.05±0.2^a^	1.14±0.17^a^	1.48±0.13
apoB100 (mmol/L) ($\bar{x}$±SD)	0.88±0.23^a^	0.82±0.14^a^	0.94±0.27	0.75±0.16

Gout: primary gout, including acute gout flare and intercritical gout; AG: acute gout flare, IG: intercritical gout, HC: healthy control subjects, $\bar{x}$±SD: mean ± standard deviation, BMI: Body Mass Index, sUA: serum uric acid; GLU: serum glucose; WBC: white blood cell counts; GR: neutrophile granulocytecounts; LY: lymphocyte counts; Mo: monocyte counts; ESR: erythrocyte sedimentation rate, CRP: reactive protein, TG: triglycerides, TC: Total Cholesterol, HDL: high-density lipoprotein; LDL: low-density lipoprotein; VLDL: very low-density lipoprotein; apoA1: apolipoprotein A1; apoB100: apolipoprotein B100; one-way ANOVA, T test or Mann Whitney test. *p*<0.05 was considered to denote statistical significance

^a^:in comparison with HC group

^b^:in comparison with IG group.

### RNA extraction

For RNA purification, we used TRIzol reagent (Invitrogen, Gran Island, NY, USA) according to the manufacturer’s instructions followed by the application of PBMCs to RNeasy spin columns (Qiagen, Venlo, Limburg, Netherlands). RNA quantity and quality were measured by NanoDrop ND-1000. RNA integrity was assessed by standard denaturing agarose gel electrophoresis or Agilent 2100 Bioanalyzer.

### LncRNA Microarray

Samples of age- and sex-matched six gout patients and six healthy controls were selected for microarray analysis. The chip used Arraystar Human LncRNA Microarray V4.0 (Arraystar, Rockville, MD, USA), which can detect about 40,173 lncRNAs and 20,730 coding transcripts. Sample labeling and array hybridization were performed according to the Agilent One-Color Microarray-Based Gene Expression Analysis protocol (Agilent Technology). Briefly, mRNA was purified from total RNA after removal of rRNA (mRNA-ONLY™ Eukaryotic mRNA Isolation Kit, Epicentre). Then, each sample was amplified and transcribed into fluorescent cRNA along the entire length of the transcripts without 3’ bias utilizing a random priming method (Arraystar Flash RNA Labeling Kit, Arraystar). The labeled cRNAs were then purified by RNeasy Mini Kit (Qiagen). An amount of 1 μg of each labeled cRNA was fragmented by adding 5 μL of 10 × blocking agent and 1 μL of 25 × fragmentation buffer, and then, we heated the mixture at 60°C for 30 min. Finally, 25 μL 2 × GE Hybridization buffer was added to dilute the labeled cRNA. A volume of 50 μL of hybridization solution was dispensed into the gasket slide and assembled to the lncRNA expression microarray slide. The slides were incubated for 17 hours at 65°C in an Agilent Hybridization Oven. The hybridized arrays were washed, fixed, and scanned using the Agilent DNA Microarray Scanner (part number G2505C).

Agilent Feature Extraction software (version 11.0.1.1) was employed to analyze the acquired array images. Quantile normalization and subsequent data processing were performed by the Gene Spring GX v12.1 software package (Agilent Technologies). Differentially expressed lncRNAs and mRNAs with statistical significance between the two groups were identified through P-value (P-value calculated from unpaired t-test; P < 0.05) filtering. Differentially expressed lncRNAs and mRNAs between the two samples were identified through Fold Change (FC > 2) filtering.

### GO and pathway analysis

Gene ontology (GO) analysis (www.geneontology.org) was used to investigate biological functions based on differentially expressed coding genes [15]. This analysis classifies functions according to the three following aspects: biological process, cellular component and molecular function. Fisher’s exact test was applied to classify the GO category. The P-value denotes the significance of GO term enrichment in the deregulated expressed genes. A lower P-value indicated a higher significance of the GO term (P-value < 0.05).

Pathway analysis was applied to assess the differentially expressed coding genes according to the Kyoto Encyclopedia of Genes and Genomes (KEGG), BioCarta, and Reactome (http://www.genome.jp/kegg/) [16]. The P-value (EASE-score, Fisher-P-value or Hypergeometric-P-value) indicates the significance of the pathway correlated with the conditions. P < 0.05 was considered statistically significant.

### LncRNA-mRNA co-expression network

The lncRNA-mRNA co-expression network identifies interactions between differentially expressed mRNAs and differentially expressed lncRNA. The normalized signal intensities of specific expression levels of mRNAs and lncRNAs were the basis of this construct. To formulate the lncRNA-mRNA co-expression network used here, we applied Pearson’s correlations to calculate the statistically significant associations.

### Quantitative real-time polymerase chain reaction (qRT-PCR) amplification

Four significantly more highly expressed lncRNAs (TCONS_00004393, ENST00000566457, NR_003542, and ENST00000430770) and four significantly more lowly expressed lncRNAs (NR_026756, TCONS_00003286, TCONS_00017125, and NR_104125) were randomly selected from the microarray results to detect their expression levels in all 96 samples (including 12 samples previously used for microarray experiment). The information of eight lncRNAs is listed in S1 Table. Extracted total RNA was reverse-transcribed to cDNA with random primer using SYBR Green RT reagents (Bio-Rad, USA). In brief, the RT reaction was performed for 60 min at 37°C followed by 60 min at 42°C, using oligo (dT) and random hexamers. PCR amplifications were performed by using SYBR Green Universal Master Mix. These reactions were performed in duplicate containing 2×concentrated Universal Master Mix, 1 μL of template cDNA, and 100 nM of primers in a final volume 12.5 μL, followed by analysis in a 96-well optical reaction plate (Bio-Rad). All reactions were performed in triplicate. The PCR results were quantified by the 2^-ΔΔct^ method against β-actin and GAPDH for normalization. The primer sequences used in the validation of lncRNAs in this paper were are listed in Table 2.

**Table 2:** Primer sequences used in the validation of lncRNAs.

gene name	Forward primers sequence (5’-3’)	Reverse primers sequence (5’-3’)
β-actin	GAGCTACGAGCTGCCTGACG	GTAGTTTCGTGGATGCCACAG
GAPDH	ATCGCCCACTTGATTTTGG	GGATTTGGTCGTATTGGGCG
TCONS_00004393	GCTGCCCAGGTGGTCTTCATG	GCCTCACAGTTGCTCATCGTCAG
ENST00000566457	CCCACGACCCCTGAGCAGAG	ACAGAGCCGAGCAGAGGAATGG
NR_026756	GGACATCCTTGCCATATCCTGCTG	GCCAGAACCAACTGCCACTCAC
TCONS_00003286	CCAGGGACCAAGTAAGCCATTGC	CCCTTCTTGGCATCTGCTTCTCTG
NR_003542	TGCTGTGTGGCCCTGTTTGC	TCCATCCCGCTGGCTTAGAAGG
ENST00000430770	GGAGATGTGATCGGTAAGGTGGTG	CTCCTAGAGGCTTCCTGGACCAC
TCONS_00017125	TCTGCGTAAGTCCCCGTAGGC	TCTGCCGAAGCCAATTCCATCATC
NR_104125	GGGAGCAGGAAGCTGGGTAATTG	AAGGGATGTGCCACTTAGGAGGAG

### Statistical analysis

Quantitative data approximating a normal distribution were expressed as the mean ± standard deviation, and non-normal distribution were expressed as median (interquartile range). Statistical analysis of demographics, clinical, and laboratory indicators were compared using independent samples t‑test, Mann‑Whitney U-test, and one‑way ANOVA, followed by the LSD post-hoc test. Correlations were calculated using Spearman’s rank correlation test. Receiver-operating characteristic (ROC) analysis was used to evaluate the power of each candidate biomarker. All statistical tests were performed using SPSS 17.0. P-values < 0.05 were considered statistically significant.

## Results

### LncRNA and mRNA profiles differ among primary patients and healthy control subjects

**Fig 1:**
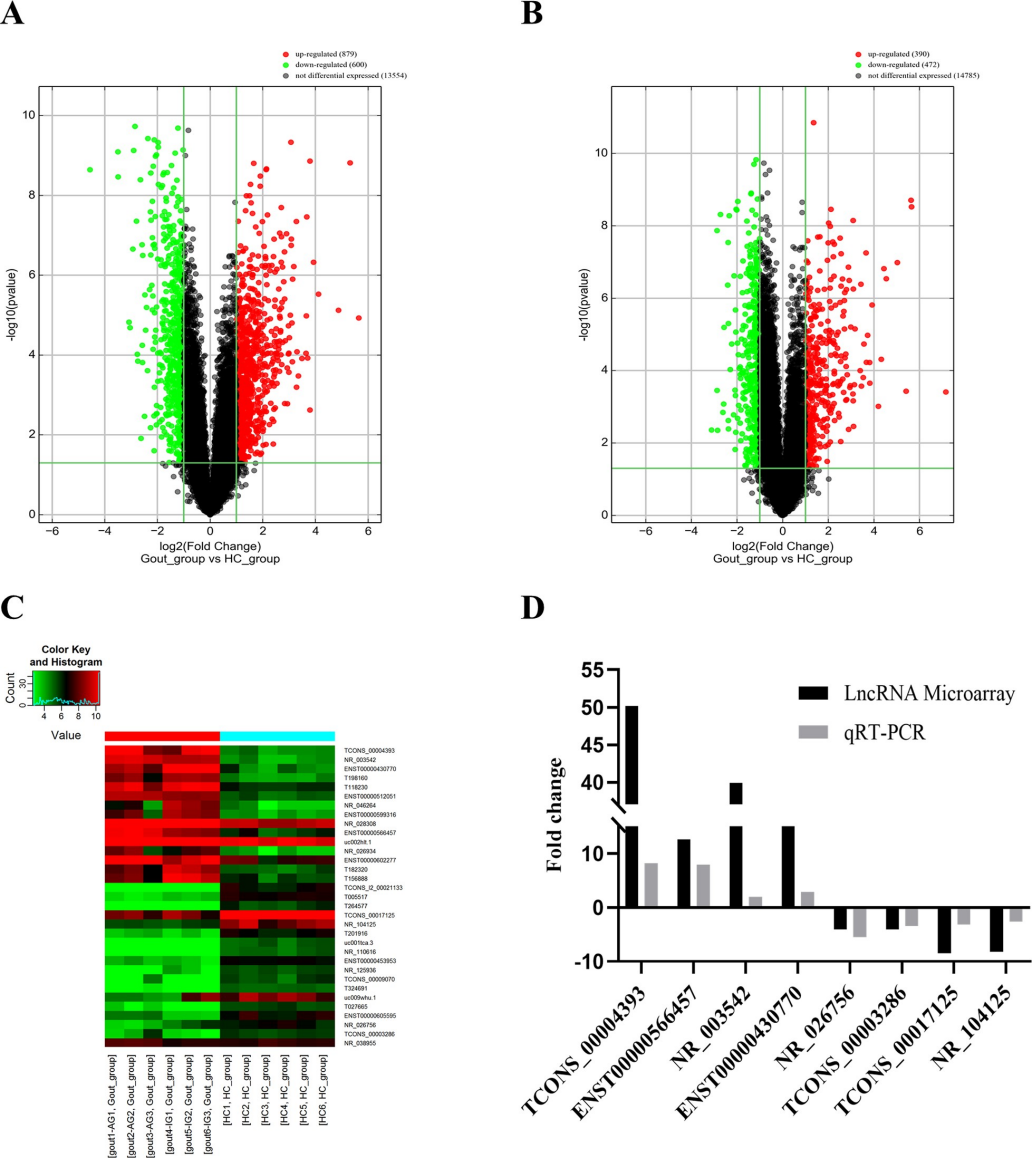
Expression profiles of lncRNAs or mRNAs in gout patients compared with healthy controls. **(A)** Volcano plot of differentially expressed lncRNAs. **(B)** Volcano plot of differentially expressed mRNAs. The horizontal green line represents a P-value of 0.05, and vertical green lines represent 2.0-fold changes up and down. X-axes is the fold change values (log2 scaled), and Y-axes is the P-values (log10 scaled). **(C)** Heatmap of 32 significantly differentially expressed lncRNAs. AG (acute gout flare) patients group: AG1, AG2, AG3; IG (intercritical gout) patients group: IG1, IG2, IG3; HC (healthy control) group: HC1, HC2, HC3. Red and green colors represent more highly expressed and more lowly expressed genes, respectively. **(D)** Relative expression levels of 8 lncRNAs in 6 gout patients and 6 healthy control subjects. The Y-axis is the ratio of the relative expression level of lncRNA in the gout group to the healthy control group.

Human LncRNA Microarray V4.0 was used to detect lncRNAs in PBMCs from 6 gout patients (including three acute gout flare and three intercritical gout patients) and 6 healthy control subjects. Volcano plot analysis was then applied for the identification of differences in lncRNAs (Fig 1A) and mRNAs (Fig 1B) from two group populations. 879 lncRNAs and 390 mRNAs were more highly expressed, whereas 600 lncRNAs and 472 mRNAs were with lower expression levels in the gout patients than in the control subjects. And the microarray data has been uploaded into a public database (accession number GES160170). Heatmap showed the expression patterns of 32 significantly different lncRNAs (Fig 1C). According to the criteria of FC > 2 and P < 0.05, the details of the top 15 more highly expressed and 15 more lowly expressed lncRNAs are presented in Table 3.

**Table 3:** The top 15 significantly more highly expressed lncRNAs and the top 15 significantly more lowly expressed lncRNAs in gout patients compared with healthy control subjects.

seqname	Gene Symbol	Fold Change	P-value	Regulation	chrom
TCONS_00004393	XLOC_002277	50.178	1.18E-05	up	chr2
NR_003542	SLED1	39.92	1.54E-09	up	chr4
ENST00000430770	RP11-136K14.1	29.429	7.57E-06	up	chr6
T198160	G045706	17.371	2.99E-06	up	chr2
T118230	G027906	15.254	4.75E-07	up	chr15
ENST00000512051	RP11-701P16.1	13.876	1.39E-09	up	chr4
NR_046264	FLJ38576	13.869	0.002389317	up	chr4
ENST00000599316	AC006129.1	13.042	0.000119015	up	chr19
NR_028308	BRE-AS1	12.749	3.47E-08	up	chr2
ENST00000566457	CTD-3247F14.2	12.605	1.05E-05	up	chr8
uc002hlt.1	D63785	12.495	9.04E-05	up	chr17
NR_026934	LOC152225	11.23	0.000121995	up	chr3
ENST00000602277	RP6-99M1.3	10.224	0.000334299	up	chrX
T182320	G041906	9.854	8.30E-05	up	chr19
T156888	G036187	9.736	0.000639856	up	chr17
ENST00000605595	RP11-1109F11.3	0.168	5.71E-05	down	chr12
T027665	G006235	0.167	1.73E-07	down	chr1
uc009whu.1	DQ592442	0.162	0.012256563	down	chr1
T324691	G076030	0.16	4.08E-09	down	chr7
TCONS_00009070	XLOC_003974	0.149	0.000142366	down	chr4
NR_125936	LOC102723828	0.147	9.57E-05	down	chr4
ENST00000453953	RP13-297E16.5	0.144	4.43E-08	down	chrX
NR_110616	LINC01355	0.138	1.87E-10	down	chr1
uc001tca.3	BC044741	0.134	7.53E-10	down	chr12
T201916	G046635	0.131	2.22E-07	down	chr2
NR_104125	IFNG-AS1	0.122	2.07E-05	down	chr12
TCONS_00017125	XLOC_007918	0.118	1.51E-05	down	chrX
T264577	G061035	0.089	3.45E-09	down	chr4
T005517	G001113	0.088	8.13E-10	down	chr1
TCONS_l2_00021133	XLOC_l2_011118	0.042	2.29E-09	down	chr4

Top 15 significantly more highly expressed lncRNAs and more lowly expressed lncRNAs.

Seqname: sequence name. P value: P value calculated from unpaired t-test. Fold change: the absolute ratio (no log scale) of normalized intensities between two groups (Gout vs HC). Chr: chromosome number from which the lncRNA is transcribed.

To validate our results independently and determine the role of lncRNAs in gout patients, eight differentially expressed lncRNAs (TCONS_00004393, ENST00000566457, NR_003542, ENST00000430770, NR_026756, TCONS_00003286, TCONS_00017125, and NR_104125) and the 12 samples previously used for microarray experiment were selected for qRT-PCR analyses. The results of the qRT-PCR showed similar trends to those observed in the microarray data. In detail, the expression levels of TCONS_00004393, ENST00000566457, NR_003542, and ENST00000430770 were higher in gout group than those in the healthy control group. The expression levels of NR_026756, TCONS_00003286, TCONS_00017125, and NR_104125 were lower in the gout group than in the healthy control group (Fig 1D).

### GO analysis

**Fig 2:**
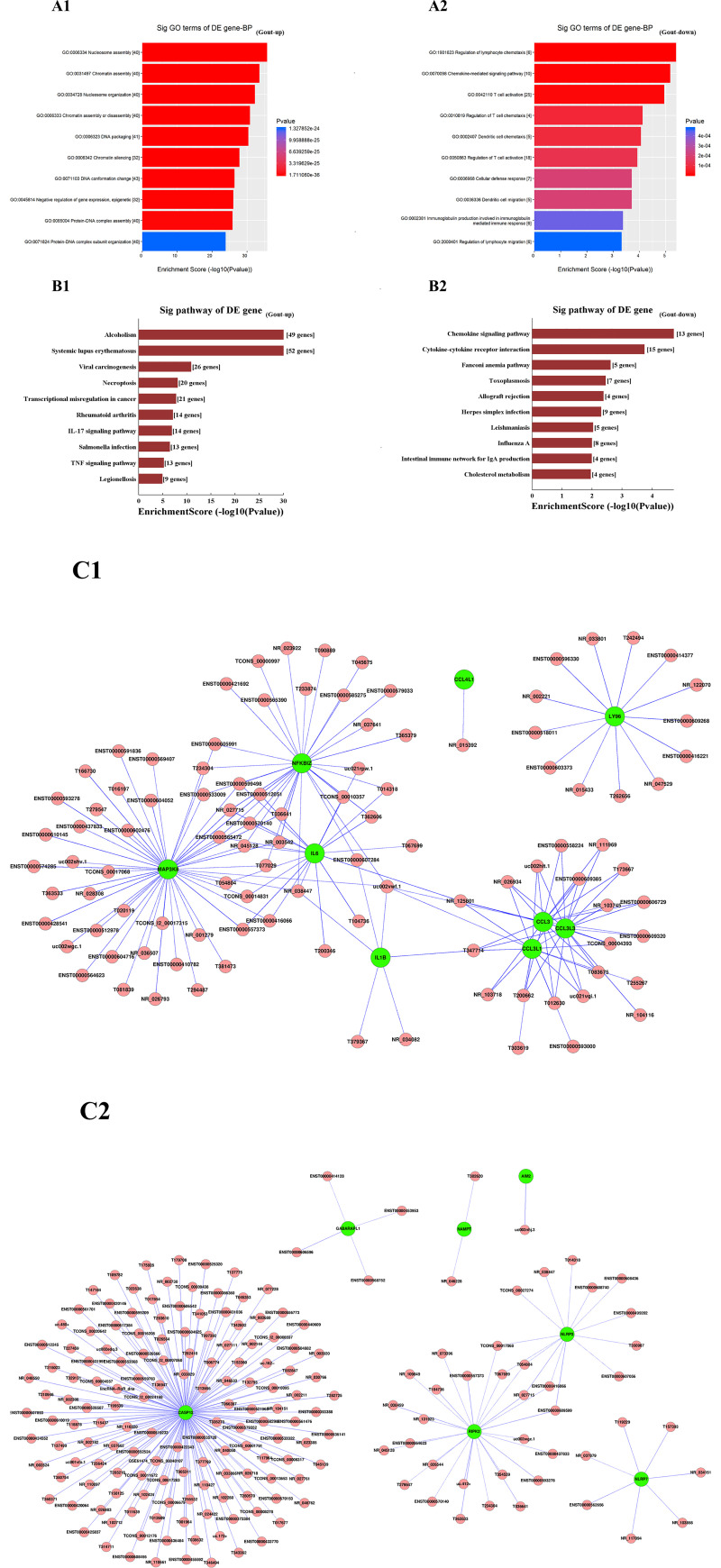
Bioinformatics analysis. **(A)** Gene ontology analyses of differentially expressed mRNAs according to the biological process (BP). **(B)** KEGG pathways analysis of differentially expressed mRNAs. The top 10 enriched GO or KEGG terms of more highly expressed mRNAs **(A1, B1)** and more lowly expressed mRNAs **(A2, B2)** are shown. The y-axis represents the most significantly enriched pathways, and the x-axis represents their scores (negative logarithm of P-value). **(C)** IncRNA-mRNA co-expression network. **(C1)** Ninety lncRNAs interacted with nine mRNAs in the meaningful “TOLL-like receptor” signaling pathway. **(C2)** One hundred and eighty lncRNAs interacted with seven mRNAs in the “NOD-like receptor” signaling pathway.

GO analysis was performed to gain insight into the potential functions of lncRNAs in the PBMCs of patients with gout. Differentially expressed mRNAs from the microarray analysis were classified into different functional categories based on the biological processes (BP) of the gene ontology. The number of significantly enriched GO terms that indicated more highly expressed and more lowly expressed mRNAs in gout were 1096 and 307, respectively. The GO analysis showed that compared with the healthy control group, the more highly expressed mRNAs in the gout group were mainly involved in “nucleosome assembly”, “chromatin silencing”, “nucleosome organization”, “chromatin assembly”, “chromatin assembly or disassembly”, and “negative regulation of gene expression, epigenetic”; the more lowly expressed mRNAs were mainly involved in “regulation of lymphocyte chemotaxis”, “chemokine-mediated signaling pathway”, “T-cell activation”, “regulation of T-cell chemotaxis”, “dendritic cell chemotaxis”, etc. (Fig 2A; top10 GOs).

### KEGG pathway analysis

KEGG Pathway analysis was used to investigate the involved biological pathways of the differentially expressed mRNAs. The number of pathway terms that the more highly expressed mRNAs in the gout group were 43 and 31, respectively. The KEGG pathway analysis revealed that compared with the healthy control group, the more highly expressed mRNAs in the gout group mainly participated in “IL-17 signaling pathway”, “TNF signaling pathway”, “Alcoholism”, “Systemic lupus erythematosus”, and “Rheumatoid arthritis”, “Viral carcinogenesis”, “Salmonella infection”, etc., whereas the more lowly expressed mRNAs were significantly involved in “Chemokine signaling pathway”, “Cytokine-cytokine receptor interaction”, “Fanconi anemia pathway”, and “Cholesterol metabolism” (Fig 2B; top10 pathways).

### LncRNA-mRNA co-expression network

The lncRNA-mRNA coexpression network may provide potential interplay between mRNAs and lncRNAs, and therefore, the differentially expressed lncRNAs and mRNAs between gout and healthy control groups were used to draw co-expression network using the Cytoscape program. Previous studies have demonstrated that TOLL-like receptor (TLR) signaling and NOD-like receptor signaling are involved in gouty arthritis development [8]. The significantly enriched KEGG pathway analysis found that the differentially expressed lncRNAs co-expressed mRNAs included the TLR signaling (hsa0460) and NOD-like receptor signaling (hsa0461), we focused on the mechanisms of gene regulation by lncRNAs in the TLR signaling and NOD-like receptor signaling pathways (Fig 2C). The network indicated that 180 lncRNAs interacting with 7 mRNAs participated in the “NOD-like receptor” signaling pathway, and 90 lncRNAs interacting with 9 mRNAs participated in the TLR signaling pathway.

### Expression levels of eight lncRNAs in gout patients and healthy control subjects

**Fig 3:**
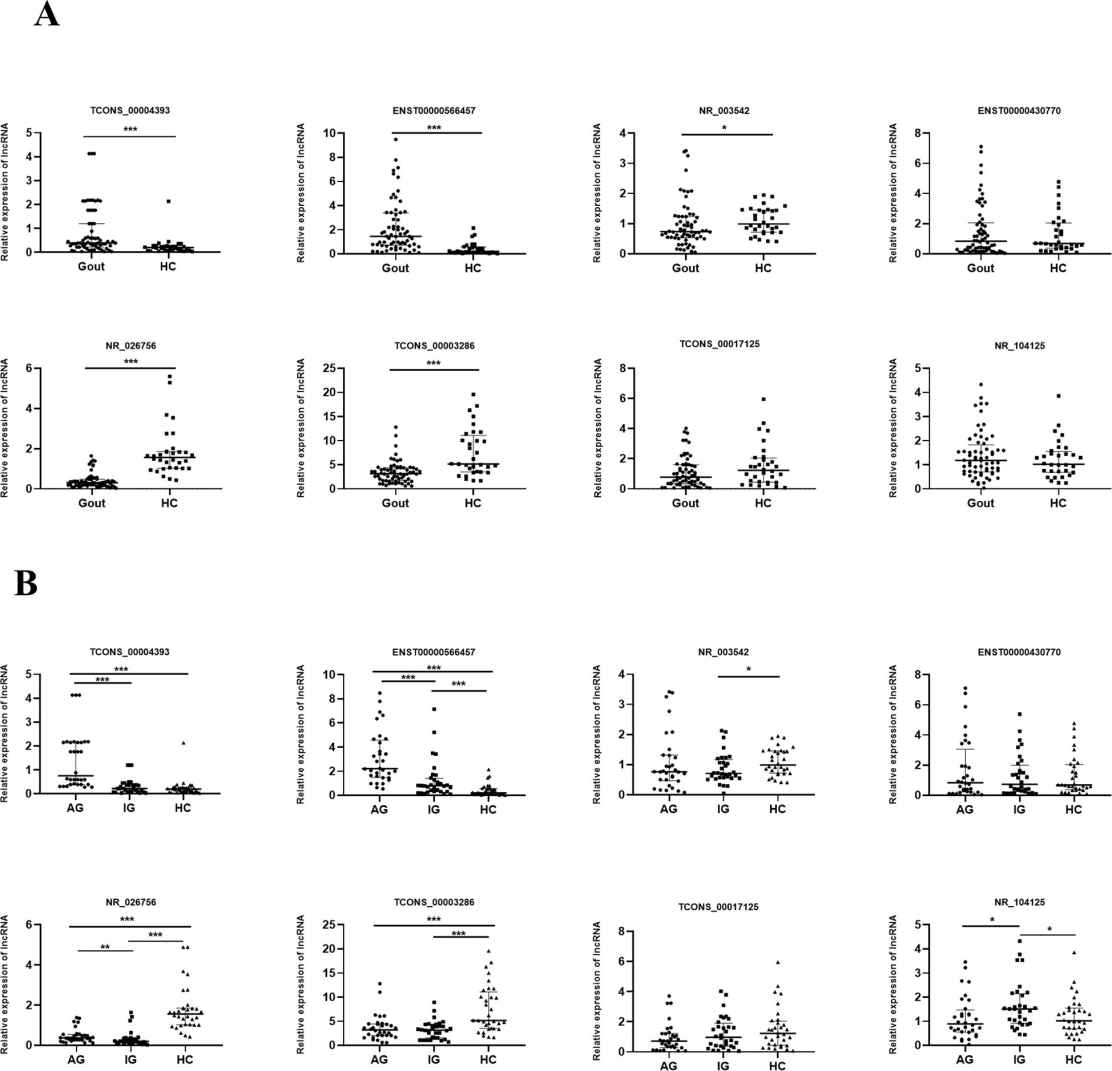
Expression levels of eight lncRNAs in gout patients and healthy control subjects. **(A)** Relative expression levels of 8 lncRNAs in 64 gout patients and 6 healthy control subjects. **(B)** Relative expression levels of 8 lncRNAs in 32 acute gout patients, 32 intercritical gout patients and 32 healthy control subjects. qRT-PCR indicates quantitative real-time polymerase chain reaction. Gout: primary gout, including acute gout and intercritical gout, AG: acute gout flare, IG: intercritical gout, HC: healthy control subjects, P < 0.05, * * P < 0.01, * * * P < 0.001.

To identify the most clinically applicable biomarkers and determine the role of lncRNAs in gout patients, eight lncRNAs expression levels were measured by qRT-PCR in 96 samples (32 acute gout flare patients, 32 intercritical gout patients and 32 healthy control subjects). Six of the eight lncRNAs expression levels from qRT-PCR analysis were in mutual agreement with the results obtained from the microarray analysis, including TCONS_00004393, ENST00000566457, NR_026756, TCONS_00003286, ENST00000430770, and TCONS_00017125. As can be seen in Fig 3A, the expression levels of TCONS_00004393 and ENST00000566457 in the GOUT group were significantly higher than those in the healthy control group (P < 0.05). The expression levels of NR_026756, TCONS_00003286, and NR_003542 were significantly lower than those of the healthy control group (P < 0.05). The expression levels of ENST00000430770, TCONS_00017125, and NR_104125 were not significantly statistically different between the two groups.

Moreover, we distinguished the expression of lncRNAs in acute gout flare, intercritical gout, and healthy control groups (Fig 3B). Significant differences were observed among the three groups in the levels of TCONS_00004393, ENST00000566457, NR_003542, NR_026756, TCONS_00003286, and NR_104125). Further study found that the TCONS_00004393 and ENST00000566457 levels in the acute gout flare group were much higher than those in the intercritical gout and healthy control group (P < 0.05), also higher expression in the acute gout flare group than that in the intercritical gout group (P < 0.05). Levels of NR_026756 and TCONS_00003286 were significantly higher in the healthy control group than in the acute gout flare and intercritical gout groups (P < 0.05). The expression levels of NR_003542 in the intercritical gout group were much lower than those in the acute gout flare and healthy control groups (P < 0.05), and NR_104125 in the intercritical gout group were much higher than those in the acute gout flare and healthy control groups (P < 0.05).

### Associations of eight lncRNAs expression levels with laboratory data in patients with gout

**Fig 4:**
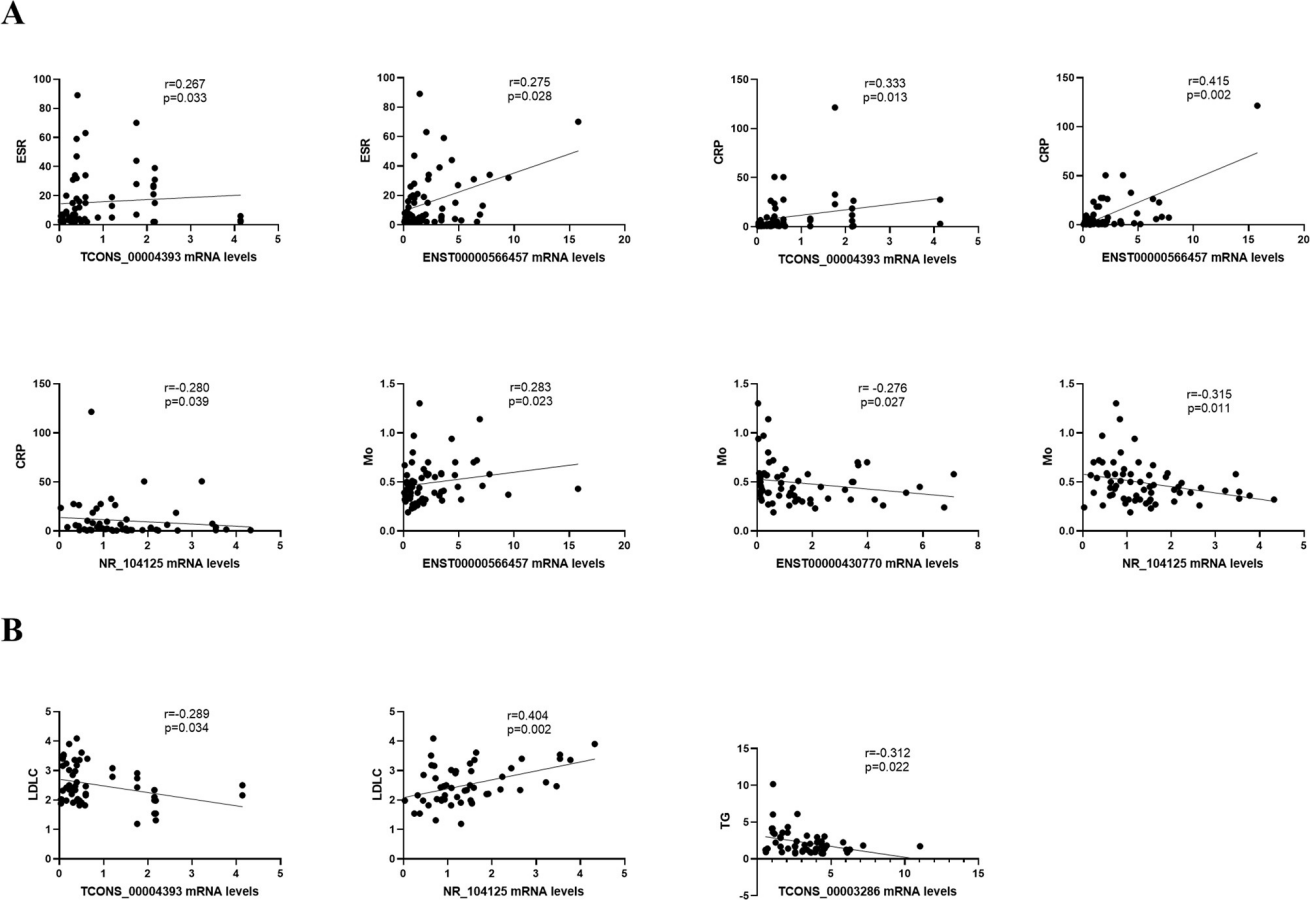
Correlations between the expression level of lncRNAs and clinical characteristics of gout analyzed with Spearman’s coefficient. **(A)** The expression level of lncRNAs were related to laboratory inflammation indicators. **(B)** The expression level of lncRNAs were related to laboratory metabolic indicators.

We evaluated the correlation between the expression level of lncRNAs and clinical indicators from the aspects of inflammation (Fig 4A) and metabolism (Fig 4B). Expressions of TCONS_00004393 and ENST00000566457 were positively related with ESR and CRP (For ESR; R = 0.267, P < 0.05; R = 0.275, P < 0.05; for CRP: R = 0.333, P < 0.05; R = 0.415, P < 0.01), and the expression of NR_104125 was negatively related with CRP (R = -0.280, P < 0.05). The expression levels of ENST00000566457, ENST00000430770 and NR_104125 are significantly related with Mo (R = 0.283, P < 0.05; R = -0.276, P < 0.05; R = 0.315, P < 0.05; respectively). LDLC was negatively related to the expression of the TCONS_00004393 (R = -0.289, P < 0.05), and positively related with expression of the NR_104125 (R = 0.404, P < 0.01). TG was negatively associated with the expression of the TCONS_00003286 (R = -0.312, P < 0.05). However, the expression levels of NR_003542, NR_026756, and TCONS_00017125 have no good correlation with any clinical parameters of gout patients. Besides, the expression levels of these eight lncRNAs were not significantly correlated with sUA, GLO, WBC, GR, LY, TC, HDL, LDL, apoA1, and apoB100.

### Diagnostic value of the five lncRNAs for gout

In recent years, many studies have established that IncRNAs can serve as novel diagnostic markers for a variety of diseases [9–11]. Thus, we sought to determine whether the differentially expressed lncRNAs identified in our microarray analysis could potentially serve as diagnostic biomarkers for gout.

**Fig 5:**
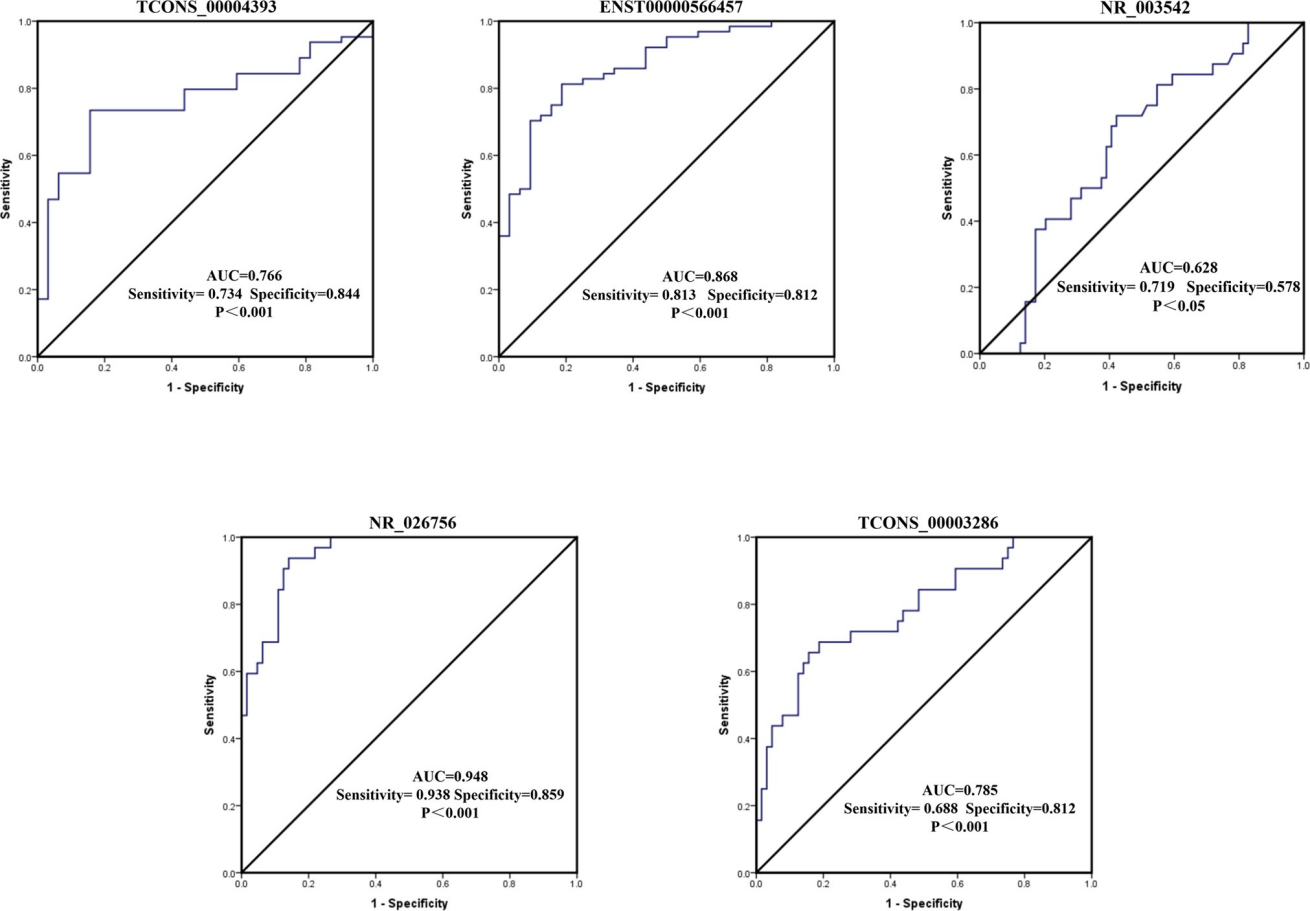
Assessment of the diagnostic value of the lncRNAs for gout. Receiver operating characteristic (ROC) curve analysis of five differently expressed lncRNAs risk scores in gout and healthy control subjects. AUC = area under the curve.

The ROC curves of five significantly differentially expressed lncRNAs (TCONS_00004393, ENST00000566457, NR_003542, NR_026756, and TCONS_00003286) in gout patients were established to estimate their diagnostic value of them in gout. The results indicated that the levels of NR_026756 and ENST00000566457 in PBMCs of gout patients and the healthy controls distinctive. As can be observed in Fig 5, the highest area under the curve (AUC) was that of NR_026756 [AUC = 0.948; 95% confidence interval (CI), 0.914–0.983; P < 0.0001; sensitivity = 93.80%, specificity = 85.90%], followed by ENST00000566457 (AUC = 0.868; 95% CI, 0.807–0.929; P <0.0001; sensitivity = 81.30%; specificity = 81.20%). In addition, the AUC of TCONS_00004393, NR_003542, and TCONS_00003286 for diagnosing gout were 0.766, 0.628, and 0.785, correspondingly; 95% CI were 0.679–0.852 (P < 0.0001), 0.529–0.727 (P < 0.05), 0.707–0.863 (P < 0.0001), respectively.

## Discussion

Although the study of lncRNAs is a hot topic, the role of lncRNAs in the pathogenesis of gout is just beginning to be investigated. Fully exploring the role of these lncRNAs in gout would provide new insights into pathogenesis of gout. With the technology of microarray analysis, we demonstrated here for the first time the expression profiles of human lncRNAs and mRNAs in primary gout. Compared with healthy people, there are differentially expressed lncRNAs in PBMC of primary gout. Furthermore, GO and KEGG pathway analysis of the differentially expressed mRNAs revealed some of the potential functions and pathways related to the pathogenesis of gout. By analyzing the correlation between 8 lncRNAs and main laboratory data of gout, we found that the differentially expressed lncRNAs may be related to the inflammation and lipid metabolism of gout. Finally, two lncRNAs (ENST00000566457 and NR_026756) had the potential to distinguish gout patients from healthy people, which may be involved in gout pathogenesis. Collectively, our results provide novel insights into the mechanism of gout development.

LncRNAs are a new class of non-coding RNAs larger than 200 nucleotides that have attracted much scientific attention in recent medical studies. Previously, the involvement of lncRNAs in immune cell development has been reported, including dendritic cell differentiation, T-cell activation, granulocytic differentiation, inhibition of T-cell proliferation, Th1-cell differentiation, regulation of interferon gamma (IFN-γ) expression, regulation of CD4+ Th2 lymphocyte migration, and CD4+ helper T lymphocyte differentiation [17–20]. Moreover, lncRNAs have been recognized as powerful regulators of numerous genes and pathways in the pathogenesis of inflammatory and autoimmune diseases, including SLE, RA, T1DM, MS, autoimmune thyroid disease, Sjögren’s syndrome, psoriasis, and Crohn’s disease [17, 21–25].

Gout is an autoinflammatory arthropathy. Accumulating evidence indicates that genetic factors, environmental triggers and immune dysregulation might be involved in gout development. However, the concrete pathogenesis of gout is still unclear. The present study was undertaken to investigate the differentially expressed lncRNAs and mRNAs in gout patients compared to healthy controls. Designed for the global profiling of human lncRNAs and protein-coding transcripts, the lncRNA microarray V4.0 system was used here to screen the different lncRNAs in gout patients to distinguish them from those in healthy controls. By comparing lncRNA and mRNA expression profiles of gout patients and controls, we found that there were 879 more highly expressed lncRNAs and 600 more lowly expressed lncRNAs, and 390 more highly expressed and 472 more lowly expressed mRNAs in the gout patients. QRT-PCR further confirmed the presence of eight differentially expressed lncRNAs. These eight lncRNAs showed consistent expression trends in the 12 samples tested in the original microarray assay, indicating that our microarray data results are highly reliable. These data may be of significance in future gout pathophysiology studies and facilitate the determination of the potential of lncRNAs in PBMCs to be used as novel non-invasive biomarkers for gout diagnosis. Our results improved our understanding of the molecular mechanisms of genes and lncRNAs in gout.

Go and KEGG pathway analyses were performed to obtain insight into the potential functions of the differentially expressed mRNAs and to improve our understanding of the mechanisms of gout development. In our results, the biological functions of more highly expressed and more lowly expressed genes were very different in gout. The more highly expressed genes were mainly involved in DNA or protein-DNA regulation, whereas the more lowly expressed genes were predominantly involved in inflammation and immunity. Previous studies have established that the TLR and NOD-like receptor signaling pathways were involved in the pathogenesis of gout inflammation [3, 4]. Interestingly, our pathway analysis found that IL-17 signaling, Systemic lupus erythematosus, Alcoholism, Viral carcinogenesis, and Salmonella infection pathways were enriched in gout patients, except for the TLR and NOD-like receptor signaling pathway. These data indicated that multiple signaling pathways associated with inflammation and metabolism were involved in gout pathogenesis.

In this study, 8 lncRNAs were verified by qRT-PCR in 64 gout patients and 32 healthy controls, and it was found that 2 lncRNAs were inconsistent with the microarray results, which may be related to sample size or individual differences. It is known that more active inflammation exists in patients with acute gout flare. Accumulating evidence suggests that lncRNAs were involved in the expression of inflammatory related genes, inflammatory signal transduction and other biological processes by acting as guidance, signal, decoy, and cytoskeleton molecules [26]. TCONS_00004393 and ENST00000566457 in acute gout flare group were highly expressed in intercritical gout group and healthy control group. Among them, the expression level of TCONS_00004393 was positively correlated with ESR and CRP, and ENST00000566457 was positively correlated with ESR, CRP and Mo, indicating that they may be related to the inflammatory response of acute gout flare. In addition, Noren et al. found that the expression level of ENST00000566457 in the elderly decreased significantly compared with young people [27]. Previous studies have shown that NLRP3 inflammasome was activated during aging [28]. Under this premise, we speculate that ENST00000566457 may be more highly expressed in the elderly, which is contrary to the experimental results of Noren et al. Interestingly, in the predicted LncRNA-mRNA co-expression network, TCONS_00004393 and ENST00000566457 were not found to participate in the "NOD-like receptor" signaling and TLR signaling pathways. Therefore, we speculate that ENST00000566457 may be involved in the "IL-17 signaling pathway", "TNF signaling pathway" and other pathways by directly regulating inflammatory factors or acting as a competitive endogenous RNA to regulate inflammatory factors, as shown by the results of the KEGG pathway, leading to acute gout flare [7, 29]. Of course, we need to do further research to reveal the mechanism of action of TCONS_00004393 and ENST00000566457 in acute gout flare.

Previous studies have shown that lncRNA is not only involved in inflammation and immune regulation, but also in metabolic regulation [30]. We know that gout is a metabolic disease, so whether lncRNA can participate in the occurrence and development of gout by regulating the metabolic response. By analyzing the correlation between the expression levels of 8 lncRNAs and the laboratory data of gout patients, we found that TCONS_00003286 was negatively correlated with the lipid metabolism index (TG). LDLC was negative and positive correlations with TCONS_00004393 and NR_104125, respectively. It suggests that these differentially expressed lncRNAs may be involved in the regulation of lipid metabolism in patients with gout. However, there were no significant correlation between lncRNA and sUA, GLU, WBC, GR, LY, TC, HDL, VDLE, apoA1 and apoB100, which may be related to the small sample size of our study or the various pathways involved in different lncRNAs. In short, the metabolic regulation mediated by lncRNAs may play a key role in the development and progression of gout.

In recent years, increasing evidence has suggested that lncRNAs can serve as novel diagnostic markers for many diseases. For example, a previous report showed that a circulating lncRNA OTTHUMT00000387022 from monocytes can be used as a novel biomarker for coronary artery disease [31]. Circulating lncRNA-HULC may be a candidate serum tumor marker for the early diagnosis of gastric cancer and for monitoring its progression and prognosis [32]. However, there are no reports regarding the use of lncRNAs as biomarkers for gout. In this study, the expression levels of TCONS_00004393 and ENST00000566457 in the gout group were significantly higher than those in the healthy control group. While the expression levels of NR_026756, TCONS_00003286, and NR_003542 were significantly lower than the ones in the healthy controls. The diagnostic values of these 5 lncRNAs were further analyzed by ROC curves. As known, ROC curve is developed in an analytical method that reflects the sensitivity and specificity of disease diagnosis indicators, with sensitivity% as the ordinate and (100%-specificity%) as the abscissa. The area under the ROC curve (AUC) indicates the diagnostic efficacy of the indicator. The value of AUC ranges from 0.5 to 1.0. Values close to 1.0 show better diagnostic efficacy. ROC analysis revealed that the AUC of ENST00000566457, NR_026756 were more than 0.8, which had excellent sensitivity (81.30% and 93.80%) and specificity (81.20% and 85.90%) in distinguishing gout patients from healthy controls. It indicates that ENST00000566457 and NR_026756 have great potential as biomarkers of gout. However, larger sample size is needed to confirm our results.

In summary, our study provides comprehensive lncRNA and mRNA profiles for gout patients. The current study provides the first demonstration that the interplay between lncRNAs and mRNA may be involved in the pathogenesis of gout, especially acute gout flare. Differentially expressed lncRNAs may be involved in the inflammation and lipid metabolism of gout. More importantly, we found that TCONS_00004393 and ENST00000566457 may be associated with acute gout pathogenesis. ENST00000566457 and NR-026756 may have diagnostic and therapeutic potential in gout.

## Supporting information

S1 Table Information of eight lncRNAs used in this study. (XLSX)

## References

[1] Dalbeth N, Merriman TR, Stamp LK. Gout. Lancet. 2016;388(10055):2039–2052. Epub 2016 Apr 21. 10.1016/S0140-6736(16)00346-9 27112094

[2] Miao Z, Li C, Chen Y, Zhao S, Wang Y, Wang Z, et al Dietary and lifestyle changes associated with high prevalence of hyperuricemia and gout in the Shandong coastal cities of Eastern China. J Rheumatol. 2008;35: 1859–1864. 18634142

[3] Martinon F, Pétrilli V, Mayor A, Tardivel A, Tschopp J. Gout-associated uric acid crystals activate the NALP3 inflammasome. Nature. 2006 3 9;440(7081):237–41. Epub 2006. 10.1038/nature04516 16407889

[4] So AK, Martinon F.Inflammation in gout: mechanisms and therapeutic targets. Nat Rev Rheumatol. 2017 11;13(11):639–647. 10.1038/nrrheum.2017.155 28959043

[5] Lee JS, Kwon OC, Oh JS, Kim YG, Lee CK, Yoo B, et al Clinical features and recurrent attack in gout patients according to serum urate levels during an acute attack. Korean J Intern Med. 2020;35(1):240–248. Epub 2019 Jan 28. 10.3904/kjim.2018.205 30685959 PMC6960048

[6] Ulitsky I, Bartel DP. lincRNAs: genomics, evolution, and mechanisms. Cell. 2013 7 3;154(1):26–46. 10.1016/j.cell.2013.06.020 23827673 PMC3924787

[7] Ernst EH, Nielsen J, Ipsen MB, Villesen P, Lykke-Hartmann K. Transcriptome Analysis of Long Non-coding RNAs and Genes Encoding Paraspeckle Proteins During Human Ovarian Follicle Development. Front Cell Dev Biol. 2018;6:78 10.3389/fcell.2018.00078 30087896 PMC6066568

[8] Aune TM, Spurlock CF 3rd. Long non-coding RNAs in innate and adaptive immunity. Virus Res. 2016;212:146–60. 10.1016/j.virusres.2015.07.003 26166759 PMC4706828

[9] Yu Y, Zhang W, Li A, Chen Y, Ou Q, He Z, et al Association of Long Noncoding RNA Biomarkers With Clinical Immune Subtype and Prediction of Immunotherapy Response in Patients With Cancer. JAMA Netw Open. 2020;3(4):e202149 10.1001/jamanetworkopen.2020.2149 32259264 PMC7139278

[10] Zhang M, Cheng L, Zhang Y. Characterization of Dysregulated lncRNA-Associated ceRNA Network Reveals Novel lncRNAs With ceRNA Activity as Epigenetic Diagnostic Biomarkers for Osteoporosis Risk. Front Cell Dev Biol. 2020;8:184 10.3389/fcell.2020.00184 32296700 PMC7136400

[11] Zhou M, Zhao H, Wang X, Sun J, Su J. Analysis of long noncoding RNAs highlights region-specific altered expression patterns and diagnostic roles in Alzheimer’s disease. Brief Bioinformatics. 2019;20(2):598–608. 10.1093/bib/bby021 29672663

[12] Min HK, Kim HR. Does normouricemic status in acute gouty arthritis really reflect a normal status? Consider confounders of serum levels of urate. Korean J Intern Med. 2020;35(1):62–64. Epub 2020 Jan 2. 10.3904/kjim.2019.423 31935321 PMC6960043

[13] Wallace SL, Robinson H, Masi AT, Decker JL, McCarty DJ, Yü TF. Preliminary criteria for the classification of the acute arthritis of primary gout. Arthritis Rheum. 1977;20:895–900. 10.1002/art.1780200320 856219

[14] Neogi T, Jansen TL, Dalbeth N, Fransen J, Schumacher HR, Berendsen D, et al 2015 Gout classification criteria: an American College of Rheumatology/European League Against Rheumatism collaborative initiative. Ann Rheum Dis. 2015;74:1789–98. 10.1136/annrheumdis-2015-208237 26359487 PMC4602275

[15] Bursill D, Taylor WJ, Terkeltaub R, Kuwabara M, Merriman TR, Grainger R, et al Gout, Hyperuricemia, and Crystal-Associated Disease Network Consensus Statement Regarding Labels and Definitions for Disease Elements in Gout. Arthritis Care Res (Hoboken). 2019; 71(3): 427–434. 29799677 10.1002/acr.23607 PMC6252290

[16] Huntley RP, Harris MA, Alam-Faruque Y, Blake JA, Carbon S, Dietze H, et al A method for increasing expressivity of Gene Ontology annotations using a compositional approach. BMC Bioinformatics. 2014;15:155 10.1186/1471-2105-15-155 24885854 PMC4039540

[17] Kanehisa M1, Goto S, Sato Y, Kawashima M, Furumichi M, Tanabe M. Data, information, knowledge and principle: back to metabolism in KEGG. Nucleic Acids Res. 2014;42(Database issue):D199-205. 10.1093/nar/gkt1076 PMC3965122 24214961

[18] Sigdel KR, Cheng A, Wang Y, Duan L, Zhang Y. The Emerging Functions of Long Noncoding RNA in Immune Cells: Autoimmune Diseases. J Immunol Res. 2015;2015:848790 10.1155/2015/848790 26090502 PMC4451983

[19] Ying L, Huang Y, Chen H, Wang Y, Xia L, Chen Y, et al Downregulated MEG3 activates autophagy and increases cell proliferation in bladder cancer. Mol Biosyst. 2013;9(3):407–11. 10.1039/c2mb25386k 23295831

[20] Gomez JA1, Wapinski OL, Yang YW, Bureau JF, Gopinath S, Monack DM, et al The NeST long ncRNA controls microbial susceptibility and epigenetic activation of the interferon-γ locus. Cell. 2013;152(4):743–54. 10.1016/j.cell.2013.01.015 23415224 PMC3577098

[21] Collier SP, Henderson MA, Tossberg JT, Aune TM. Regulation of the Th1 genomic locus from Ifng through Tmevpg1 by T-bet. J Immunol. 2014;193(8):3959–65. 10.4049/jimmunol.1401099 25225667 PMC4185266

[22] Chatenoud L. Immune therapies of autoimmune diseases: are we approaching a real cure? Curr Opin Immunol. 2006;18(6):710–7. 10.1016/j.coi.2006.09.004 17011768

[23] Xu F, Jin L, Jin Y, Nie Z, Zheng H. Long noncoding RNAs in autoimmune diseases. J Biomed Mater Res Part A. 2019: 107A: 468–475. 10.1002/jbm.a.36562 30478988

[24] Li Z, Li X, Jiang C, Qian W, Tse G, Chan MTV, et al Long non-coding RNAs in rheumatoid arthritis. Cell Prolif. 2018;51(1). 10.1111/cpr.12404 PMC6620844 29110355

[25] Zhang Y, Xu YZ, Sun N, Liu JH, Chen FF, Guan XL, et al Long noncoding RNA expression profile in fibroblast-like synoviocytes from patients with rheumatoid arthritis. Arthritis Res Ther. 2016;18(1):227 10.1186/s13075-016-1129-4 27716329 PMC5053204

[26] Gao Y, Li S, Zhang Z, Yu X, Zheng J. The Role of Long Non-coding RNAs in the Pathogenesis of RA, SLE, and SS. Front Med (Lausanne). 2018;5:193. 30018955 10.3389/fmed.2018.00193 PMC6038710

[27] Noren HN, Evans MK. Age and poverty status alter the coding and noncoding transcriptome. Aging (Albany NY). 2019;11:1189–1203. 30779705 10.18632/aging.101823 PMC6402526

[28] Cordero MD, Williams MR, Ryffel B. AMP-Activated Protein Kinase Regulation of the NLRP3 Inflammasome during Aging. Trends Endocrinol Metab. 2018; 29: 8–17. 10.1016/j.tem.2017.10.009 29150317

[29] Xu YT, Leng YR, Liu MM, Dong RF, Bian J, Yuan LL, et al MicroRNA and long noncoding RNA involvement in gout and prospects for treatment. Int Immunopharmacol. 2020; 87: 106842 10.1016/j.intimp.2020.106842 32738598

[30] Xu J, Cao X. Long noncoding RNAs in the metabolic control of inflammation and immune disorders. Cell Mol Immunol. 2019; 16(1): 1–5. 10.1038/s41423-018-0042-y 29795339 PMC6318285

[31] Cai Y, Yang Y, Chen X, Wu G, Zhang X, Liu Y, et al Circulating ’lncRNA OTTHUMT00000387022’ from monocytes as a novel biomarker for coronary artery disease. Cardiovasc Res. 2016;112(3):714–724. 10.1093/cvr/cvw022 26857419

[32] Jin C, Shi W, Wang F, Shen X, Qi J, Cong H, et al Long non-coding RNA HULC as a novel serum biomarker for diagnosis and prognosis prediction of gastric cancer. Oncotarget. 2016 9;7(32):51763–51772. 10.18632/oncotarget.10107 27322075 PMC5239513

